# A Case of Type 2 Protein S Deficiency Presenting as Cerebral Venous Thrombosis (CVT) in an 18-Year-Old Female

**DOI:** 10.7759/cureus.28221

**Published:** 2022-08-20

**Authors:** Ankush Agarwal S, Jennie Santhanam, Arun K, Sruthi Degapudi, Subramaniyan K

**Affiliations:** 1 General Medicine, Sri Ramaswamy Memorial (SRM) Medical College Hospital and Research Centre, Chengalpattu, IND

**Keywords:** cerebral venous thrombosis (cvt), oral anticoagulation, acquired protein s deficiency, qualitative defect, hereditary protein s deficiency

## Abstract

Here, we report a case of cerebral venous thrombosis in an 18-year-old female. On evaluation, she was found to have type 2 protein S deficiency, which is the rarest form of protein S deficiency and is also known as a qualitative defect. Protein S is primarily synthesized by hepatocytes and undergoes vitamin K-dependent gamma-carboxylation. Mature protein S circulates in two states: free and bound to the complement component C4b-binding protein (C4b-BP). The free form of protein S acts as a cofactor for activated protein C. This case is unique as here, there is a qualitative effect that is responsible for the manifestations.

## Introduction

Protein S deficiency is an autosomal dominant condition due to mutations in the *PROS1* gene, a large gene on chromosome 3. It is associated with an increased risk of thromboembolism. Protein S is primarily synthesized by hepatocytes and undergoes vitamin K-dependent gamma-carboxylation [1]. Mature gamma-carboxylated protein S circulates in two states: free and bound to the complement component C4b-binding protein (C4b-BP). The free form comprises 30-40% of total protein S and is the only form of protein S that has cofactor activity for activated protein C [1,2]. Total protein S values differ with age, whereas free protein S values remain relatively constant.

Protein S was named after Seattle, Washington, where it was first discovered and purified. It is a vitamin K-dependent glycoprotein and serves as a cofactor for activated protein C, which in turn inactivates procoagulant factors Va and VIIIa, reducing thrombin generation [3]. Protein S also serves as a cofactor for activated protein C in enhancing fibrinolysis and can directly inhibit prothrombin activation via interactions with other coagulation factors [4-8]. Protein S deficiency interferes with the normal control mechanism and thereby increases the risk of thrombosis.

## Case presentation

An 18-year-old female presented to the outpatient department (OP) with complaints of headache for five days, holocranial (left > right), continuous type with no aggravating or relieving factors. It was associated with two episodes of vomiting, two days prior. Vomitus was nonprojectile, non-blood-tinged, non-bilious, and containing food particles. Following this, she developed abnormal sensation over the left half of her body, including her face, trunk, upper limb, and lower limb. She denied having visual disturbances, seizures, fever, neck pain, loss of consciousness, fall, or trauma to the head. She denied having similar complaints in the past. She had no previous hospital admissions.

Her family history was insignificant, and she denies taking any drugs or treatment for any ailments. She is unmarried and has attained menarche at the age of 14 with her last menstrual cycle starting 18 days prior. She was not sexually active and was not on any medications such as oral contraceptive pills.

She was conscious and oriented to time, place, and person. Her vitals were blood pressure (BP) of 90/60 mm of mercury, pulse rate of 92/minute, respiratory rate of 20/minute with an oxygen saturation of 97% under room air, and afebrile. Pallor was present with no other abnormality in her general physical examination.

On examination of her central nervous system, higher mental functions were found to be normal; she showed decreased perception to touch when examining her trigeminal nerve (maxillary and mandibular divisions). All other cranial nerves were found to be normal. Her motor system examination showed no abnormality with normal reflexes. Her sensory system examination revealed a deficit over the left side with decreased pain perception and tactile sensation on the left upper limb and lower limb. Joint position sense was intact. The cerebellar function was intact. There was no abnormality found in the examination of her respiratory, abdominal, and cardiovascular systems.

She was taken up for a CT brain, which showed “hyperdense inferior sagittal and left transverse sinus.” A possibility of cerebral venous thrombosis was considered. Her lab parameters showed hemoglobin of 6.9 g/dl, mean corpuscular volume (MCV) of 54 fl, mean corpuscular hemoglobin (MCH) of 13 pg, mean corpuscular hemoglobin concentration (MCHC) of 24 g/dl, total WBC count of 11,440 cells/cumm with neutrophilic predominance of 61.3%, and lymphocytes of 33%. At presentation, she was found to have mild transaminitis, which resolved subsequently. Her renal parameters and electrolytes were found to be normal. Prothrombin time (PT), activated partial thromboplastin time (aPTT), and international normalized ratio (INR) were also within normal limits. Antinuclear antibody (ANA) and rheumatoid factor (RF) were negative.

**Figure 1:**
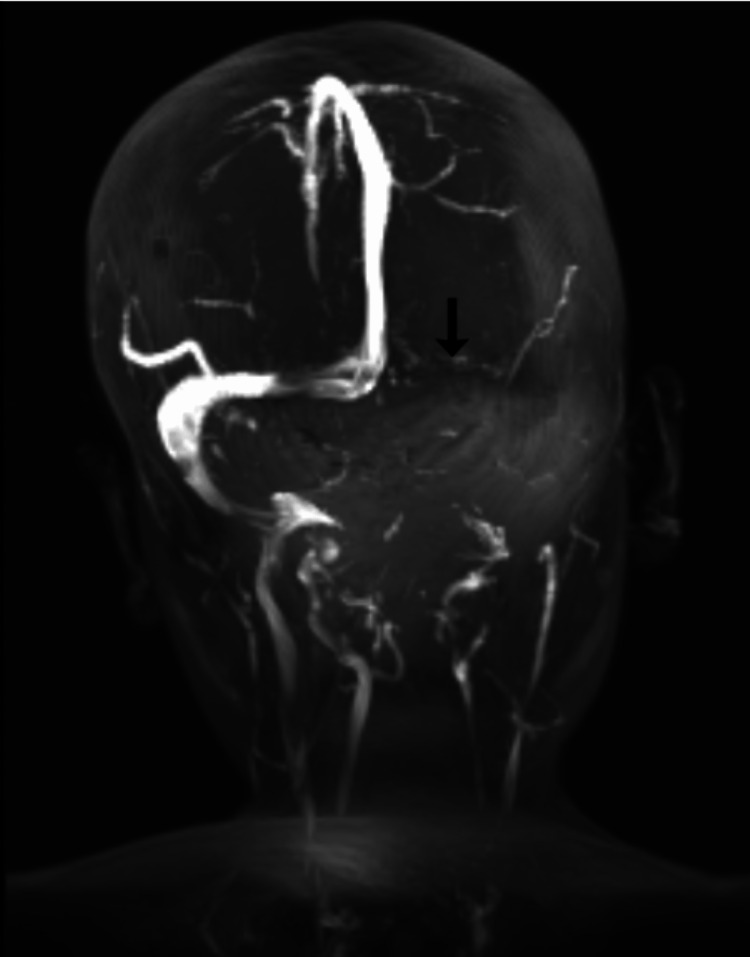
MRV image at presentation Thrombosis of the left transverse dural venous sinus (black arrow); MRV: magnetic resonance venography

MRI brain with magnetic resonance angiography (MRA) and magnetic resonance venography (MRV) (Figure 1) showed “early acute venous infarct in the right thalamus with thrombosis of deep cerebral veins and dural venous sinuses and multiple acute lacunar infarcts in bilateral centrum semiovale and corona radiata.”

The patient was started on anticoagulant treatment. Other causes of cerebral venous thrombosis such as dehydration, smoking, and infection were ruled out. Procoagulation studies were delayed as acute thrombosis can give a false reading of decreased values.

**Figure 2:**
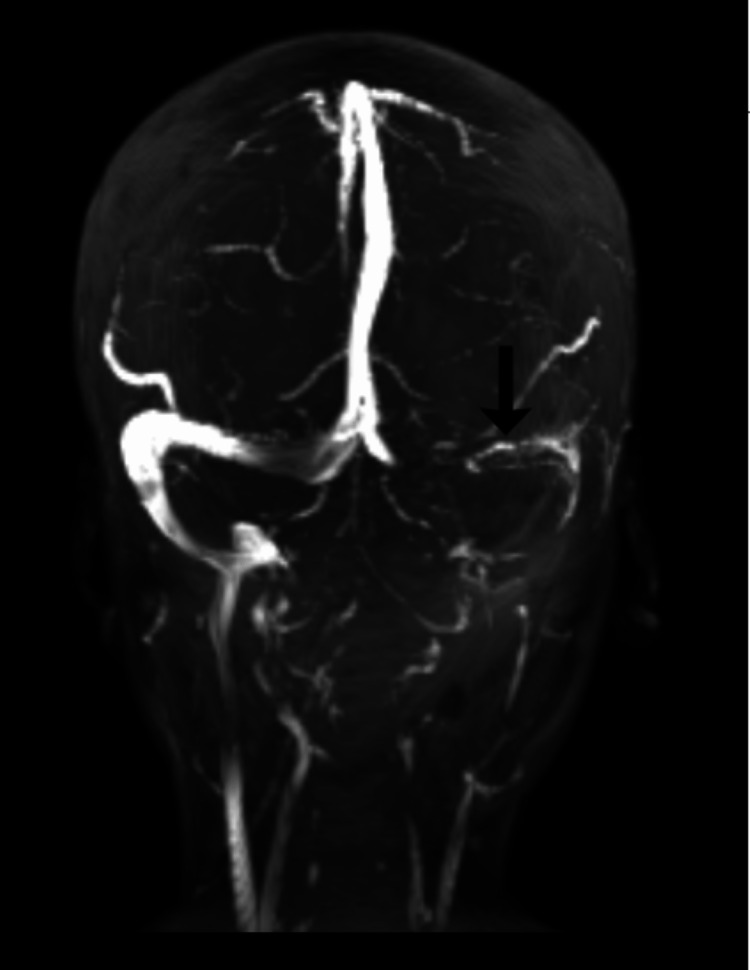
MRV image at follow-up Partial recanalization of the lateral aspect of the left transverse sinus noted (black arrow) when compared to the previous image (Figure 1); MRV: magnetic resonance venography

Repeat MRI was done six months post initiation of treatment, and the findings were “discontinuous, serpiginous, flow-related signals at places in the left transverse sinus, features suggestive of chronic cerebral venous sinus thrombosis with partial recanalization” (Figure 2).

Procoagulation studies (Table 1) were done at this point after stopping anticoagulation therapy for two weeks; it showed lupus anticoagulant screen ratio of 1.13; homocysteine levels were normal with a value of 8.73 µmol/L, and IgG and IgM phospholipid antibody was found to be normal with values of 3.57 IgG phospholipid unit (GPL) U/ml and 5.35 IgM phospholipid unit (MPL) U/ml, respectively. Her IgG and IgM beta 2 glycoprotein levels were also found to be normal. She had slightly elevated functional antithrombin activity with a value of 122% (normal range: 80-120%) and a decrease in functional protein S activity with a value of 6% (normal range: 55-123%). Her free protein S antigen values were found to be normal, i.e., 71% (normal range: 60-140%). Her functional protein C values were normal. Genetic testing revealed no mutation in factor V Leiden and prothrombin gene.

**Table 1:** Procoagulation Study GPL: IgG phospholipid unit; MPL: IgM phospholipid unit; SGU: serum IgG antibody unit; SMU: serum IgM antibody unit

Test	Result	Normal Range
Lupus Anticoagulation Screen Ratio	1.13	<1.20
Serum Homocysteine	8.73 µmol/L	4.44-13.56
Serum Phospholipid Antibody, IgG	3.57 GPL U/ml	<12.00
Serum Phospholipid Antibody, IgM	5.35 MPL U/ml	<12.00
Serum Beta 2 Glycoprotein, IgG	1.21 SGU	<20.00
Serum Beta 2 Glycoprotein, IgM	3.42 SMU	<20.00
Free Protein S Antigen	71%	60-140%
Protein S Functional/Activity	6%	55-123%
Functional Protein C	107%	70-140%
Functional Antithrombin Activity	122%	80-120%
Factor V Leiden Mutation Analysis	Not Detected	Not Detected
Prothrombin Gene Mutation	Not Detected	Not Detected

She was continued on her anticoagulation therapy and significantly improved in due course of time with complete regain of her sensory deficits.

## Discussion

Recurrent venous thrombosis used to be recognized on a clinical basis with no apparent cause until the mid-1960s. After which, antithrombin III (AT-III) deficiency was recognized as a possible cause by Egeberg in 1965 [8]. Protein C and protein S deficiencies were recognized almost 16 years later by Griffin et al. and Comp et al. In 1981, Griffin et al. [9] reported familial protein C deficiency in association with venous thromboembolism, and in 1984, Comp and Esmon [10] reported protein S deficiency in six patients; five were with onset between 15 and 27 years of age. These studies demonstrated that the congenital deficiencies of protein S, protein C, and AT-III are inherited in an autosomal dominant fashion.

In our particular case, the patient is of young age with no risk factor for thrombotic event. Her family history was uneventful, which posed a dilemma in the clinical diagnosis of any of the above-mentioned deficiencies. This warranted an evaluation in detail, and on further review of literature, she was found to have type 2 protein S deficiency, which is considered to be one of the rarest forms.

Protein S deficiencies have been classified into three types [11]; type 1 is the classical type of inherited deficiency. There is reduced total protein S, free protein S, and protein S function. Type 2 represents a qualitative defect and is one of the rarest forms of this deficiency where only the protein S function is reduced as witnessed in our case. Type 3 represents the selective reduction of free protein S and functional protein S with normal total protein S values.

With regard to type 2 protein S deficiency, in a case series of 118 French patients with thromboembolism associated with protein S deficiency, 26 had a serine to proline substitution at amino acid 460 (the Heerlen polymorphism), which affects protein S metabolism [12,13]. The low free plasma protein S may result from increased binding of the abnormal protein S to C4b-binding protein [14,15]. Most patients with type 2 protein S deficiency do not manifest into thrombophilic episodes, but in our case, this was the only abnormality found, which could attribute to said manifestation.

## Conclusions

With this case report, we would like to highlight the importance of detailed evaluation in a young patient with thromboembolic event with no apparent cause and negative family history. Although thrombophilic events in type 2 protein S deficiency have been questioned, this case had no other alternative causes. The patient’s clinical manifestation and supportive evidence of decreased protein S activity levels support the etiology of qualitative protein S deficiency. The patient was regularly followed up and has been in good health with oral anticoagulation therapy.
